# Validation and functional annotation of expression-based clusters based on gene ontology

**DOI:** 10.1186/1471-2105-7-380

**Published:** 2006-08-15

**Authors:** Ralf Steuer, Peter Humburg, Joachim Selbig

**Affiliations:** 1 University Potsdam, Institute for Biochemistry and Biology, Karl-Liebknecht-Strasse 24-25, Haus 20, 14476 Potsdam, Germany; 2 University Potsdam, Institute for Physics, Nonlinear Dynamics Group, Am Neuen Palais 10, 14469 Potsdam, Germany; 3 Max-Planck-lnstitute of Molecular Plant Physiology, Am Mühlenberg 1, 14476 Potsdam, Germany

## Abstract

**Background:**

The biological interpretation of large-scale gene expression data is one of the paramount challenges in current bioinformatics. In particular, placing the results in the context of other available functional genomics data, such as existing bio-ontologies, has already provided substantial improvement for detecting and categorizing genes of interest. One common approach is to look for functional annotations that are significantly enriched within a group or cluster of genes, as compared to a reference group.

**Results:**

In this work, we suggest the information-theoretic concept of mutual information to investigate the relationship between groups of genes, as given by data-driven clustering, and their respective functional categories. Drawing upon related approaches (Gibbons and Roth, Genome Research 12:1574-1581, 2002), we seek to quantify to what extent individual attributes are sufficient to characterize a given group or cluster of genes.

**Conclusion:**

We show that the mutual information provides a systematic framework to assess the relationship between groups or clusters of genes and their functional annotations in a quantitative way. Within this framework, the mutual information allows us to address and incorporate several important issues, such as the interdependence of functional annotations and combinatorial combinations of attributes. It thus supplements and extends the conventional search for overrepresented attributes within a group or cluster of genes. In particular taking combinations of attributes into account, the mutual information opens the way to uncover specific functional descriptions of a group of genes or clustering result. All datasets and functional annotations used in this study are publicly available. All scripts used in the analysis are provided as additional files.

## Background

One of the common assertions in expression analysis is that genes sharing a similar pattern of expression are more likely to be involved in the same regulatory processes [1]. This proposition, commonly referred to as 'guilt-by-association', has been exploited by a large number of clustering algorithms, grouping genes into a (small) number of classes, based on the similarity of their expression profiles. While there are still many open problems associated with choosing a particular algorithm, clustering has already proven successful in a multitude of applications, such as the inference of putative functional annotations [2,3], as well as the extraction of regulatory motifs in the upstream regions of genes [4,5].

More recently, the data-centric view, i.e. based on measured expression levels alone, has been advanced to integrate additional information, such as existing functional annotations [6-9] or protein-protein interaction [10-12]. In doing so, the paramount task is to enhance the biological interpretation of the data, e.g. by identifying physiologically relevant categories, based on existing bio-ontologies, associated with a particular grouping of genes.

However, prior to such a step, it is necessary to obtain a better understanding about the specific relationship between the data generated clustering and the information contained in the functional annotation of genes. That is, to what extent does a grouping of genes reflect their functional annotation, as e.g. given in terms of the structured vocabulary provided by the gene ontology (GO) consortium? In this work, we thus investigate the relationship between groupings of genes and their respective functional categories. It will be shown that the information-theoretic concept of mutual information provides a suitable theoretical basis to address this question in a systematic way. Importantly, the mutual information holds several favorable properties and *i*) allows to give a quantitative figure of merit between a clustering result and functional annotations. *ii*) allows to identify functional trends that characterize a given clustering or grouping of genes, *iii*) allows to address and incorporate the interdependence of functional annotation terms, and *iv*) can be straightforwardly applied to the whole set of clusters, or likewise, only to a single individual cluster of group or genes.

Within this framework, we aim to extend the earlier work of Gibbons and Roth [13] and seek to investigate to what extend individual attributes are sufficient to characterize or summarize a given cluster of genes. This question is also ultimately related to the problem of detecting significantly enriched attributes within a group of genes, well covered in the literature [14-17]. We will demonstrate that in certain situations a simple search for overrepresented attributes fails to uncover the specific functional description of clustering results.

The paper is organized as follows: In the first section, a brief synopsis of the mutual information as a measure of dependency between cluster membership and annotated gene attributes is given. In the second section, we address the capabilities of individual attributes to characterize or summarize a given cluster of genes. In the following section, two major shortcomings of this approach will be pointed out: The interrelatedness of gene attributes (redundancy) and the failure of individual attributes to adequately describe a given clustering or grouping of genes. To overcome these problems, a heuristic strategy is devised that allows to detect combinatorial combinations of attributes, providing a more specific functional description of clustering results. The results are summarized and discussed in the last section.

## Results

### The mutual information

Following Gibbons and Roth [13], the *mutual information I*(*C*, *A*) provides a figure of merit between cluster membership *C *and known gene attributes *A*,

*I*(*C*, *A*) = *H*(*C*) + *H*(*A*) - *H*(*C*, *A*)       (1)

where *H*(*C*), *H*(*A*) and *H*(*C*, *A*) denote the entropies of the distributions of *C *and *A *and the joint entropy of *C *and *A *respectively.

H(C)=−∑ip(Ci)log⁡p(Ci)     (2)
 MathType@MTEF@5@5@+=feaafiart1ev1aaatCvAUfKttLearuWrP9MDH5MBPbIqV92AaeXatLxBI9gBaebbnrfifHhDYfgasaacH8akY=wiFfYdH8Gipec8Eeeu0xXdbba9frFj0=OqFfea0dXdd9vqai=hGuQ8kuc9pgc9s8qqaq=dirpe0xb9q8qiLsFr0=vr0=vr0dc8meaabaqaciaacaGaaeqabaqabeGadaaakeaacqWGibascqGGOaakcqWGdbWqcqGGPaqkcqGH9aqpcqGHsisldaaeqbqaaiabdchaWjabcIcaOiabdoeadnaaBaaaleaacqWGPbqAaeqaaOGaeiykaKIagiiBaWMaei4Ba8Maei4zaCMaemiCaaNaeiikaGIaem4qam0aaSbaaSqaaiabdMgaPbqabaGccqGGPaqkaSqaaiabdMgaPbqab0GaeyyeIuoakiaaxMaacaWLjaWaaeWaceaacqaIYaGmaiaawIcacaGLPaaaaaa@4956@

The mutual information is a general measure of dependency between two (or more) variables [18-20] and can be interpreted as a 'distance' between the hypothesis of statistical independence and the actual joint probability distribution [18]. A more detailed review concerning its mathematical properties and the estimation from finite data was given elsewhere [19,20]. Importantly, the mutual information *I*(*C, A*) is zero *if and only if *the two variables, here the gene attributes *A *and the cluster membership *C*, are statistically independent. As will be demonstrated below, this property incloses and extends conventional approaches, such as finding significantly enriched annotations associated with a group of genes.

**Figure 1:**
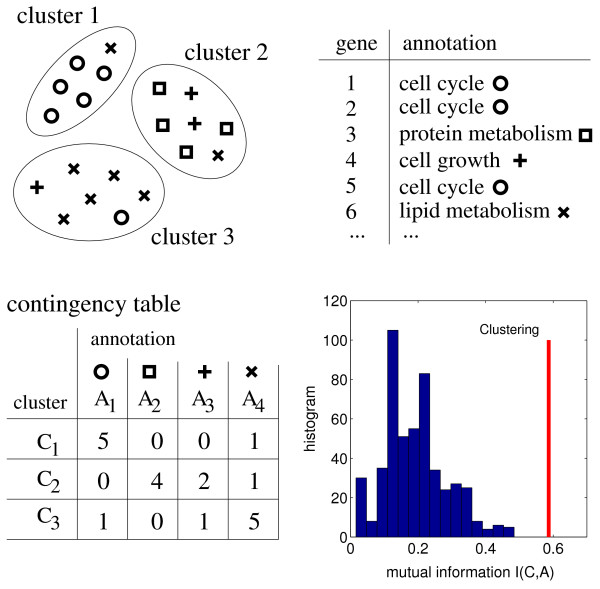
Validating clustering results by the mutual information: A schematic example. Each gene is uniquely assigned to one functional category *A*_*i *_and grouped into cluster *C*_*j *_by a given clustering algorithm. The joint probabilities can be straightforwardly estimated from the associated contingency table and the mutual information is calculated according to Eq. (1). To assess how related the clustering is to the annotation, the value of the mutual information is compared to random assignments of genes to cluster number, i.e. each gene is randomly assigned to a cluster, preserving the total number of genes within each cluster, but destroying all possible relationship between the clustering and the functional annotation. The lower right plot shows the mutual information, compared to an ensemble of 500 randomized assignments, In this example, the z-score, estimated according to Eq. (8), is *S *≈ 3.8. For a z-score to be deemed significant, we further require that no random assignment results in a mutual information equal or larger that the tested annotation. Note that, though we expect the mutual information to be zero for the randomized assignments, the average estimated mutual information for randomized data has a bias towards positive values due to finite-size effects [19,20]. As a rule of thumb, to obtain reliable estimate of the mutual information the number of genes should be at least three times larger than the number of clusters or functional categories [20].

In the most simplest setting, each gene is uniquely assigned to one particular functional category *A*_*i *_and is grouped into a cluster *C*_*j *_by a given clustering algorithm. In this case the estimation of the mutual information is straightforward: One constructs a contingency table and estimates the respective probabilities from the relative frequencies of occurrence, as schematically visualized in Fig. 1.

The mutual information thus provides a systematic quantitative measure of the relationship between cluster membership and given gene attributes [13]. In particular, it opens the possibility to judge the quality of a clustering result, not based on internal measures of consistency, such as within cluster distances to inter cluster distances, but based on existing additional information.

However, in our case, the multi-functions of genes, as reflected in current annotation databases, defy the straightforward approach outlined above. In the following, we will make use of the curator-controlled annotation of *S. cerevisiae *genes, as provided by the gene ontology consortium (GO) [21].

The GO database is organized in a rooted directed acyclic graph (DAG), with three branches corresponding to the three categories 'cellular component', 'molecular function' and 'biological process'. Each gene (or rather gene product) is annotated by one or multiple GO terms along the graph. The hierarchical nature of GO implies that genes annotated with a specific node are also annotated with every ancestor of that node. Nodes closer to the root of the graph usually correspond to more abstract functional descriptions and cover more genes, while nodes farther away from the root correspond to more detailed functional descriptions. Note that the structure of GO is not necessarily a tree since each node may have multiple parents and may have multiple paths to the root of the graph [22].

The GO database, as downloaded in June 2004. already includes more than *N*_*A *_> 16000 nodes within all three branches [23]. For further numerical processing, each gene is assigned to a vector consisting of binary attributes **A **= {*A*_1_, *A*_2_,..., ANA
 MathType@MTEF@5@5@+=feaafiart1ev1aaatCvAUfKttLearuWrP9MDH5MBPbIqV92AaeXatLxBI9gBaebbnrfifHhDYfgasaacH8akY=wiFfYdH8Gipec8Eeeu0xXdbba9frFj0=OqFfea0dXdd9vqai=hGuQ8kuc9pgc9s8qqaq=dirpe0xb9q8qiLsFr0=vr0=vr0dc8meaabaqaciaacaGaaeqabaqabeGadaaakeaacqWGbbqqdaWgaaWcbaGaemOta40aaSbaaWqaaiabdgeabbqabaaaleqaaaaa@304B@} with *A*_*i *_∈ {0,1}, where *A*_*i *_= 1 indicates that the gene has been annotated with the GO term *A*_*i *_or one of its descendants.

Clearly, in such a situation, a straightforward estimation of the mutual information must inevitably fail: We would have to take into account all possible combinations of attributes *A*_*i*_, resulting in a contingency table with up to 2NA
 MathType@MTEF@5@5@+=feaafiart1ev1aaatCvAUfKttLearuWrP9MDH5MBPbIqV92AaeXatLxBI9gBaebbnrfifHhDYfgasaacH8akY=wiFfYdH8Gipec8Eeeu0xXdbba9frFj0=OqFfea0dXdd9vqai=hGuQ8kuc9pgc9s8qqaq=dirpe0xb9q8qiLsFr0=vr0=vr0dc8meaabaqaciaacaGaaeqabaqabeGadaaakeaacqaIYaGmdaahaaWcbeqaaiabd6eaonaaBaaameaacqWGbbqqaeqaaaaaaaa@3028@ columns, as illustrated in Table 1. Even though the vast majority of combinations does not occur for the genes under consideration, a direct evaluation of Eq. (1), even for just a few hundreds of different attributes, is beyond all computational and statistical means.

**Table 1:** The multi-functions of genes defy a straightforward estimation of the mutual information. Each gene is assigned to a vector of binary attributes A = {*A*_1_, *A*_2_, ..., ANA
 MathType@MTEF@5@5@+=feaafiart1ev1aaatCvAUfKttLearuWrP9MDH5MBPbIqV92AaeXatLxBI9gBaebbnrfifHhDYfgasaacH8akY=wiFfYdH8Gipec8Eeeu0xXdbba9frFj0=OqFfea0dXdd9vqai=hGuQ8kuc9pgc9s8qqaq=dirpe0xb9q8qiLsFr0=vr0=vr0dc8meaabaqaciaacaGaaeqabaqabeGadaaakeaacqWGbbqqdaWgaaWcbaGaemOta40aaSbaaWqaaiabdgeabbqabaaaleqaaaaa@304B@}, described by a number *a*_*i *_∈ [0, 2NA
 MathType@MTEF@5@5@+=feaafiart1ev1aaatCvAUfKttLearuWrP9MDH5MBPbIqV92AaeXatLxBI9gBaebbnrfifHhDYfgasaacH8akY=wiFfYdH8Gipec8Eeeu0xXdbba9frFj0=OqFfea0dXdd9vqai=hGuQ8kuc9pgc9s8qqaq=dirpe0xb9q8qiLsFr0=vr0=vr0dc8meaabaqaciaacaGaaeqabaqabeGadaaakeaacqaIYaGmdaahaaWcbeqaaiabd6eaonaaBaaameaacqWGbbqqaeqaaaaaaaa@3028@ - 1]. The contingency table to evaluate the mutual information *I*(*C*, [*A*_1_, ..., ANA
 MathType@MTEF@5@5@+=feaafiart1ev1aaatCvAUfKttLearuWrP9MDH5MBPbIqV92AaeXatLxBI9gBaebbnrfifHhDYfgasaacH8akY=wiFfYdH8Gipec8Eeeu0xXdbba9frFj0=OqFfea0dXdd9vqai=hGuQ8kuc9pgc9s8qqaq=dirpe0xb9q8qiLsFr0=vr0=vr0dc8meaabaqaciaacaGaaeqabaqabeGadaaakeaacqWGbbqqdaWgaaWcbaGaemOta40aaSbaaWqaaiabdgeabbqabaaaleqaaaaa@304B@]), taking all possible combinations into account, would thus include up to 2NA
 MathType@MTEF@5@5@+=feaafiart1ev1aaatCvAUfKttLearuWrP9MDH5MBPbIqV92AaeXatLxBI9gBaebbnrfifHhDYfgasaacH8akY=wiFfYdH8Gipec8Eeeu0xXdbba9frFj0=OqFfea0dXdd9vqai=hGuQ8kuc9pgc9s8qqaq=dirpe0xb9q8qiLsFr0=vr0=vr0dc8meaabaqaciaacaGaaeqabaqabeGadaaakeaacqaIYaGmdaahaaWcbeqaaiabd6eaonaaBaaameaacqWGbbqqaeqaaaaaaaa@3028@ columns.

		annotation						
	Cluster	*A*_1_	*A*_2_	*A*_3_	*A*_4_	...	ANA MathType@MTEF@5@5@+=feaafiart1ev1aaatCvAUfKttLearuWrP9MDH5MBPbIqV92AaeXatLxBI9gBaebbnrfifHhDYfgasaacH8akY=wiFfYdH8Gipec8Eeeu0xXdbba9frFj0=OqFfea0dXdd9vqai=hGuQ8kuc9pgc9s8qqaq=dirpe0xb9q8qiLsFr0=vr0=vr0dc8meaabaqaciaacaGaaeqabaqabeGadaaakeaacqWGbbqqdaWgaaWcbaGaemOta40aaSbaaWqaaiabdgeabbqabaaaleqaaaaa@304B@	**A**
gene 1	**1**	0	1	0	0	...	1	*a*_1_
gene 2	**2**	0	0	0	1	...	0	*a*_2_
gene 3	**1**	1	0	1	0	...	0	*a*_3_
⋮	⋮	⋮				...	⋮	⋮

To overcome this problem, Gibbons and Roth [13] suggested to approximate the total mutual information as a sum of the mutual information between clusters and each individual attribute.

I(C,[A1,...,ANA])≈∑i=1NAI(C,Ai)     (3)
 MathType@MTEF@5@5@+=feaafiart1ev1aaatCvAUfKttLearuWrP9MDH5MBPbIqV92AaeXatLxBI9gBaebbnrfifHhDYfgasaacH8akY=wiFfYdH8Gipec8Eeeu0xXdbba9frFj0=OqFfea0dXdd9vqai=hGuQ8kuc9pgc9s8qqaq=dirpe0xb9q8qiLsFr0=vr0=vr0dc8meaabaqaciaacaGaaeqabaqabeGadaaakeaacqWGjbqsdaqadiqaaiabdoeadjabcYcaSmaadmGabaGaemyqae0aaSbaaSqaaiabigdaXaqabaGccqGGSaalcqGGUaGlcqGGUaGlcqGGUaGlcqGGSaalcqWGbbqqdaWgaaWcbaGaemOta40aaSbaaWqaaiabdgeabbqabaaaleqaaaGccaGLBbGaayzxaaaacaGLOaGaayzkaaGaeyisIS7aaabCaeaacqWGjbqscqGGOaakcqWGdbWqcqGGSaalcqWGbbqqdaWgaaWcbaGaemyAaKgabeaakiabcMcaPaWcbaGaemyAaKMaeyypa0JaeGymaedabaGaemOta40aaSbaaWqaaiabdgeabbqabaaaniabggHiLdGccaWLjaGaaCzcamaabmGabaGaeG4mamdacaGLOaGaayzkaaaaaa@5241@

With

*I*(*C*, *A*_*i*_) = *H*(*C*) + *H*(*A*_*i*_) - *H*(*C*, *A*_*i*_)       (4)

Note that this approximation assumes both, independence and conditional independence between all attributes.

To illustrate this, we make use of a simple example involving just two attributes *A*_1 _and *A*_2_. One has to evaluate

*I*(*C*, [*A*_1_, *A*_2_]) = *H*(*C*) + *H*(*A*_1_, *A*_2_) - *H*(*C*, *A*_1_, *A*_2_)       (5)

Since for statistically independent attributes *H*(*A*_1_, *A*_2_) = *H*(*A*_1_) + *H*(*A*_2_), we only need to consider the last term *H*(*C*, *A*_1_, *A*_2_). Assuming conditional independence *H*(*A*_1_|*A*_2_, *C*) = *H*(*A*_1_|*C*), we obtain

*H*(*C*, *A*_1_, *A*_2_) = *H*(*C*, *A*_1_) + *H*(*C*, *A*_2_) - *H*(*C*)       (6)

Thus Eq. (5) indeed reduces to

*I*(*C*, [*A*_1_, *A*_2_]) = *I*(*C*, *A*_1_) + *I*(*C*, *A*_2_)       (7)

Given the structure of the GO database, as described above, the assumption of statistical independence is, of course, *not *fulfilled: The attributes are not statistically independent but strongly dependent on each other. In particular, any annotated attribute *A*_*i *_implies that all of its parents are also annotated. This interdependence will be considered in more detail below.

Unfortunately, Eq. (3) does likewise not allow to give an upper or lower bound on the true mutual information. It is well established that this approximation does not result in a systematic bias, i.e. in general one may not tell whether the violation of the assumptions under- or overestimates the true mutual information [18].

However, for the moment we accept Eq. (3) as a reasonable approximation of the mutual information. Based on this assumption, it was already demonstrated that clustering results and the GO annotations indeed possess a mutual information significantly different from zero [13]. Interestingly, the widely used hierarchical clustering gave results not significantly different from random assignments and was found to be far worse than other common algorithms, such as k-means.

In the following, we will draw upon these results, using the same datasets and preprocessing of the GO database as described by Gibbons and Roth [13]. but addressing slightly different questions instead.

### The case of individual attributes

Given that clustering results and the known functional annotation indeed yield a mutual information significantly different from zero, the question arises how this mutual information is distributed among the individual attributes. Are there only few attributes which correspond to and summarize the cluster? Or, on the other hand, is the observed overlap tightly embedded within the structure of the GO annotations – a combined effect of a multitude of attributes, where neither of them is sufficient to characterize a given cluster on its own?

To evaluate this, we must have a closer look on the distribution of the individual terms contributing to Eq. (3). Restricting ourselves to a k-means algorithm (see appendix), the individual mutual information *I*(*C, A*_*i*_) between the clustering *C *and all attributes was estimated.

To assess how related each each attribute is with the clustering, we evaluate randomized assignments of genes to clusters, i.e. each gene is randomly assigned to a cluster, preserving the total number of genes within each cluster. This results in the z-score

S=I(C,A)data−〈I(C,A)random〉σrandom     (8)
 MathType@MTEF@5@5@+=feaafiart1ev1aaatCvAUfKttLearuWrP9MDH5MBPbIqV92AaeXatLxBI9gBaebbnrfifHhDYfgasaacH8akY=wiFfYdH8Gipec8Eeeu0xXdbba9frFj0=OqFfea0dXdd9vqai=hGuQ8kuc9pgc9s8qqaq=dirpe0xb9q8qiLsFr0=vr0=vr0dc8meaabaqaciaacaGaaeqabaqabeGadaaakeaacqWGtbWucqGH9aqpdaWcaaqaaiabdMeajjabcIcaOiabdoeadjabcYcaSiabdgeabjabcMcaPmaaCaaaleqabaGaeeizaqMaeeyyaeMaeeiDaqNaeeyyaegaaOGaeyOeI0IaeyykJeUaemysaKKaeiikaGIaem4qamKaeiilaWIaemyqaeKaeiykaKYaaWbaaSqabeaacqqGYbGCcqqGHbqycqqGUbGBcqqGKbazcqqGVbWBcqqGTbqBaaGccqGHQms8aeaaiiGacqWFdpWCdaWgaaWcbaGaeeOCaiNaeeyyaeMaeeOBa4MaeeizaqMaee4Ba8MaeeyBa0gabeaaaaGccaWLjaGaaCzcamaabmGabaGaeGioaGdacaGLOaGaayzkaaaaaa@5AD2@

where *σ*_random _denotes the standard deviation of the estimated mutual information for the randomized data. For the z-score to he considered significant, we further require that the number of random assigments is larger that the total number of tested attributes and that tor each attribute all random assigments result in a lower mutual information.

**Figure 2:**
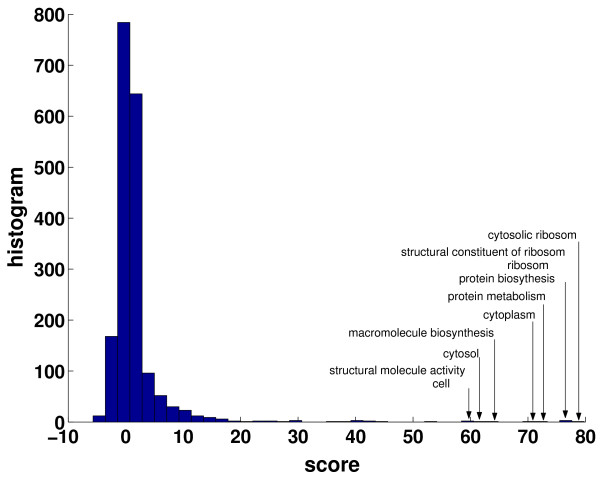
Histogram of the z-score Eq. (8) for all individual attributes. The attributes with the highest score are marked. The figure is for the cell cycle dataset of Spellman et al. [24] using the same preprocessing as described in [13] (sec appendix]. After filtering ≈ 2500 GO attributes remained for evaluation. Repeating the analysis for all datasets given in Table 2 yields similar results. The clustering was obtained using a *k*-means algorithm with Euclidean distance and *k *= 25, the results do not change significantly for different choices of *k *(tested between *k *= 5 - 30, corresponding to the region where the z-score of the mutual information is largest [13]). Note that the top scoring attributes appear to be largely redundant, i.e. a gene that is annotated to the cellular component 'cytosolic ribosome' can be intuitively expected to be also annotated to the biological process 'protein biosynthesis'. See next section for details.

Figure 2 shows a histogram of the obtained scores, estimated according to Eq. (8). for the cell cycle dataset of Spellman *et al*. [24] and *k *= 25 cluster. As can be observed the vast majority of annotated attributes shows no, or only little, significant overlap with the data-driven clustering. However, the overall distribution is highly inhomogeneous: few attributes are singled out and possess a remarkably high z-score with respect to their shuffled counterparts. The ten highest scoring attributes are indicated in Fig. 2. Likewise, we must also expect the aggregated mutual information of Eq. (3) to be dominated by only few addends of rather high value.

**Table 2:** Several datasets were used to verity the results, corresponding to different experimental setups and conditions. In each case, only the 3000 genes with highest variance were selected for further analysis. Note that this implies that the set of selected genes is (slightly) different for each dataset. For details on the preprocessing and normalization of the data see Appendix. In the following, all shown results will refer to the dataset of Spellman *et al*.[24]

Name		no. of points	Description	Ref.
Spellman *at al*.	(1998)	75	Cell Cycle	[24]
Zhu *at al*.	(2000)	26	Cell Cycle	[25]
Gasch *at al*.	(2000)	175	Various conditions	[26]

Interestingly, the highest scoring attributes do not change substantially when different datasets are considered (see Table 2 for all datasets under consideration). This indicates that different experimental conditions, corresponding here to different datasets, do not significantly influence which attributes are selected as the most descriptive for the clusters.

### Combinatorial combinations of attributes

Two essential shortcoming of our analysis have to be pointed out: First of all. Fig. 2 strongly suggests that the top scoring attributes are largely redundant, i.e. that the individual terms contributing to the aggregated mutual information of Eq. (3) are not independent.

In fact, a gene product annotated with the cellular component 'cytosolic ribosome' (GO:0005830) is necessarily also annotated to the cellular component 'ribosome' (GO:0005840). While this is a trivial consequence of the tree structure of the database (the former node being a child of the latter), other relationships between attributes are less straightforward. For example, and even without any computational assistance, gene products annotated to the cellular component 'ribosome' (GO:0005840) can mostly be expected to be also annotated to the biological process 'protein biosynthesis' (GO:0006412). However, it is worth pointing out that this, in contrast to the former example, is not an inherent consequence of the tree structure of the database, as both nodes appear within distinct, branches of the tree. More systematically, we can assess the redundancy between two selected attributes again by means of the (pair-wise) mutual information *I *(*A*_*i*_, *A*_*j*_) between two attributes *A*_*i *_arid *A*_*j*_. Table 3 gives the contingency tables for the five top scoring attributes of Fig. 2. As can be seen, the selected attributes are indeed highly interdependent.

**Table 3:** The contingency tables between the live top scoring attributes given in Fig. 2, along with the z-score *S *for the pair-wise mutual information *I*(*A*_*i*_, *A*_*j*_), estimated according to Eq. (8) with respect to 500 randomized realizations. High values of *S *indicate that both attributes are not independent, i.e. that the probability of observing such a value of the mutual information *I*(*A*_*i*_, *A*_*j*_) for statistically independent attributes *A*_*i *_and *A*_*j *_is low. Shown are the nodes: 'GO:0005830' (component: cytosolic ribosome). 'GO:0003735' (function: structural constituent of ribosome). 'GO:0005840' (component: ribosome). 'GO:0006412' (process: protein biosynthesis), and 'GO:0019538' (process: protein metabolism). Note that the contingency tables, as well as the z-score, was estimated for the full set of 6312 genes. Reducing the analysis to those 3000 genes used in the creation of Fig. 2 increases the redundancy even more.

		G0:0005830		GO:0003735		GO:0005840		GO:0006412		GO:0019538	
		**1**	**0**	**1**	**0**	**1**	**0**	**1**	**0**	**1**	**0**
GO:0005830	**1**	140	0	137	3	140	0	137	3	137	3
	**0**	0	6172	61	6111	89	6083	421	5751	709	5463
		-		**S ≈ 702**		**S ≈ 687**		**S ≈ 430**		**S ≈ 394**	

GO:0003735	**1**	137	61	198	0	196	2	198	0	198	0
	**0**	3	6111	0	6114	33	6081	360	5754	648	5466
		-		-		**S ≈ 1037**		**S ≈ 704**		**S ≈ 585**	

GO:0005840	**1**	140	89	196	33	229	0	219	10	220	9
	**0**	0	6083	2	6081	0	6083	339	5744	626	5457
		-		-		-		**S ≈ 771**		**S ≈ 649**	

GO:0006412	**1**	137	421	198	360	219	339	558	0	558	0
	**0**	3	5751	0	5754	10	5744	0	5754	288	5466
		-		-		-		-		**S ≈ 1663**	

GO:0019538	**1**	137	709	198	648	220	626	558	288	816	0
	**0**	3	5463	0	5466	0	5457	0	5466	0	5466
		-		-		-		-		-	

This, of course, points to a major drawback of the analysis: The individual attributes contributing dominantly to the sum of Eq. (3), do not represent independent information about a specific grouping of genes or clustering result. Rather, by selecting the attributes according to their individual mutual information, we explore areas in which the GO annotations are interdependent.

It should be noted that a similar situation occurs in the conventional search for overrepresented attributes within a group or cluster of genes. Again, not taking the non-independence of attributes into account will often result in a selection of mostly redundant functional annotation terms. This, of course, affects the interpretation of the results, as these attributes do not actually contribute to a characterization of the given clustering. In the following, after pointing out a second drawback of our analysis, we will thus devise a strategy that incorporates the interdependence of attributes.

### Detecting combinations of attributes

Apart from above described shortcoming, restricting ourselves to the mutual information between a given clustering and single individual attributes, entails yet another problem. While these (possibly redundant) attributes can indeed indicate a functional association between the genes gathered in a particular cluster, other, more specific, functional descriptions might be easily missed.

In most cases, it will not only be one attribute that defines or characterizes a group or cluster of genes, but rather a specific combination of attributes. Table 4 gives an illustrative example of such a situation. Here, neither of the attributes share any mutual information with the grouping of the genes into two clusters, nor is any attribute overrepresented within the two groups. However, looking at the combination of both attributes does immediately reveal that these attributes are nonetheless highly descriptive for the given cluster: Their combination does uniquely determine to which cluster a particular gene belongs, or, vice versa, which annotation coincides with a particular cluster.

**Table 4:** Combinations of GO attributes: Shown is a schematic example of 8 genes, separated into two distinct clusters (Table 4a). As can be observed neither of the two given attributes is significantly enriched within any of the cluster, resulting in a vanishing mutual information between the clustering and the annotation (see the respective contingency tables in Table 4b and 4c). However, clearly the combination of both attributes does uniquely determine both cluster. In particular, genes with the combination A = (*A*_1_, *A*_2_) = (0, 0) or (1, 1) are grouped together in the first cluster 0. while genes sharing the annotation A = (0, 1) or (1, 0) are grouped together in the second cluster.

**4a**								
gene	1	2	3	4	5	6	7	8
cluster	**0**	**0**	**1**	**0**	**1**	**1**	**1**	**0**

*A*_1_	1	0	0	0	0	1	1	1
*A*_2_	1	0	1	0	1	0	0	1

**4b**								
Cluster:		0	1					

*A*_1_	0	2	2					
	1	2	2					

**4c**								
Cluster:		0	1					

*A*_2_	0	2	2					
	1	2	2					

Obviously, the tremendous computational demands prevent to conduct an exhaustive GO-wide search for all possible combinations of attributes. To still detect relevant combinations for large-scale data, we thus devise a simple heuristic strategy: Starting with a seed attribute A_0_, the one that gives the highest mutual information *I*(*C, A*_0_), we iteratively look for attributes that result in the largest information gain, i.e. the largest increase in mutual information, when included in the list of attributes. Schematically:

initialize: **A **= *A*_0_

search ∀*i *: *I*(*C*, [**A**, *A*_*i*_]) → max ⇒ *A*_*l*_

test for significance: *I*(*C*, [**A**, Airandom
 MathType@MTEF@5@5@+=feaafiart1ev1aaatCvAUfKttLearuWrP9MDH5MBPbIqV92AaeXatLxBI9gBaebbnrfifHhDYfgasaacH8akY=wiFfYdH8Gipec8Eeeu0xXdbba9frFj0=OqFfea0dXdd9vqai=hGuQ8kuc9pgc9s8qqaq=dirpe0xb9q8qiLsFr0=vr0=vr0dc8meaabaqaciaacaGaaeqabaqabeGadaaakeaacqWGbbqqdaqhaaWcbaGaemyAaKgabaGaeeOCaiNaeeyyaeMaeeOBa4MaeeizaqMaee4Ba8MaeeyBa0gaaaaa@376B@])

update: **A **= (**A**, *A*_*l*_)

Thus, at each step a new attribute *A*_*l *_is included into the vector **A **of already selected attributes and the mutual information *I*(*C*, A) is calculated without using the approximation of Eq. (3). In this way. attributes which are highly redundant to those already included in **A **will not be selected. For example, assume that after an attribute *A*_0 _a second attribute *A*_1 _is tested. If both are fully redundant, then *H*(*A*_0_, *A*_1_) = *H*(*A*_0_) and *H*(*C*, *A*_0_, *A*_1_) = *H*(*C*, *A*_0_). Thus, according to Eq. (5), *I*(*C*, [*A*_0_, *A*_1_]) = *H*(*C*) + *H*(*A*_0_, *A*_1_) - *H*(*C*, *A*_0_, *A*_1_) ≡ *H*(*C*, *A*_0_), i.e. there is no gain in information and the attribute will not be included into **A**.

The iteration stops after a predefined maximal number of steps *l*^max ^or when no new attribute leads to a significant increase in mutual information. The latter is tested at each iteration step by comparing the result to randomized counterparts of the attribute to be included. As in the previous section, we require that the number of randomized assignments is larger that the total number of tested attributes and that no randomized assignment yields a mutual information equal or higher than the attribute that is to be included. Otherwise, the increase in mutual information is not considered significant and the iteration stops. Note that this also avoids statistical problems due to finite size effects [20]. If the vector of attributes becomes too large, a reliable estimation of the mutual information is no longer possible. In this case, the resulting values upon inclusion of a new attribute will not significantly deviate from those of randomized attributes.

Thus, instead of conducting a comprehensive search for all possible combinations, we consider only those attributes which further contribute to a characterization of a clustering result, given the already selected attributes. In this way, we avoid the inclusion of a large number of redundant attributes. Note that this procedure is reminiscent of a decision tree [29], aiming to predict the cluster assignment based on the GO annotation.

Table 5 shows the result for the previously considered dataset of Spellman *at al*. [24]. Starting with the highest scoring attribute '*cytosolic ribosome' *(GO:0005830), already depicted in Fig. 2, new attributes were iteratively included until *l*^max ^= 31, the first 20 are given in Table 5.

**Table 5:** Combinations of GO attributes selected for the dataset of Spellman et al. [24]. Starting with the highest scoring attribute '*cytosolicribosome*'. new attributes were iteratively included until *k*^max ^= 31, the first, 20 are given here. Note that the results do not depend specifically on which of the datasets was used: GO IDs that have been selected among the top 32 for *all *datasets listed in Table 2 are indicated in bold. The clustering was the same as considered above, see caption of Fig. 2 for details. Note that neither of the attributes is dedicatedly related to the cell cycle, except 'cell cycle' and 'mitosis', which were likewise found for all of the considered datasets.

rank k	GO ID	description
0	**GO:0005830**	**cytosolic ribosome (sensu Eukaryota)**
1	**GO:0005737**	**cytoplasm**
2	**GO:0007049**	**cell cycle**
3	**GO:0005634**	**nucleus**
4	**GO:0003824**	**catalytic activity**
5	**GO:0006411**	**protein metabolism**
6	GO:0016043	cell organization and biogenesis
7	**GO:0008152**	**metabolism**
8	**GO:0003676**	**nucleic acid binding**
9	**GO:0016020**	**membrane**
10	**GO:0007275**	**development**
11	GO:0009058	biosynthesis
12	**GO:0008151**	**cell growth and/or maintenance**
13	**GO:0005215**	**transporter activity**
14	**GO:0005739**	**mitochondrion**
15	GO:0006259	DNA metabolism
16	GO:0009056	catabolism
17	G0:0006519	amino acid and derivative metabolism
18	**GO:0005975**	**carbohydrate metabolism**
19	**GO:0007067**	**mitosis**
20	GO:0005488	binding

Constructing the associated contingency table of the 5 top scoring attributes, analog to Table 3, indeed reveals that the pair wise mutual information between the selected attributes is significantly lower. The respective z-scores are given in Table 6.

**Table 6:** The contingency tables of the the live top scoring attributes listed in Table 5. Note that in this case the respective scores are significantly lower, as compared to the results given in Table 3. This indicates that the respective attributes are, though not statistically independent, much less redundant than in the previous case. Shown are the nodes: 'GO:0005830' (component: cytosolic ribosome), 'GO:0005737' (component: cytoplasm). 'GO:0007049' (process: cell cycle). 'GO:0005634' (component: nucleus), 'GO:0003824' (function: catalytic activity).

		GO:0005830		GO:0005737		GO.0007049		GO:0005634		GO:0003824	
		**1**	**0**	**1**	**0**	**1**	**0**	**1**	**0**	**1**	**0**
GO:0005830	**1**	140	0	140	0	0	140	1	139	0	140
	**0**	0	6172	1108	5064	390	5782	520	5652	1153	5019
		-		**S ≈ 288**		**S ≈ 11.2**		**S ≈ 11.0**		**S ≈ 41.6**	

GO:0005737	**1**	140	1108	1248	0	124	1124	46	1202	364	884
	**0**	0	5064	0	5064	266	4798	475	4589	789	4275
		-		-		**S ≈ 21.3**		**S ≈ 33.3**		**S ≈ 72.4**	

GO:0007049	**1**	0	390	124	266	390	0	139	251	140	250
	**0**	140	5782	1124	4798	0	5922	382	5540	1013	4909
		-		-		-		**S ≈ 177.9**		**S ≈ 48.3**	

GO:0005634	**1**	1	520	46	475	139	382	521	0	193	328
	**0**	139	5652	1202	4589	251	5540	0	5791	960	4831
		-		-		-		-		**S ≈ 81.2**	

GO:0003824	**1**	0	1153	364	789	140	1013	198	960	1153	0
	**0**	140	5019	884	4275	250	4909	328	4831	0	5159
		-		-		-		-		-	

Again the results were not specific for the particular dataset. Repeating the analysis for all datasets given in Table 2 resulted in similar attributes. Those attributes that were selected among the top 32 for *all *datasets under consideration are indicated in bold in Table 5.

This again indicates that the specific experimental condition, under which the dataset was obtained (two cell cycle experiments and one alternative experiment, involving several conditions.), has no, or only little, influence over the prevailing functional annotations that characterizes the clustering of this respective dataset. This is noteworthy, as in each case different genes were selected for the analysis (see description in Table 2). Moreover, the clustering results itself were sufficiently different, i.e. this mutuality in descriptive annotations is not straightforwardly apparent on the level of clusters itself.

**Figure 3:**
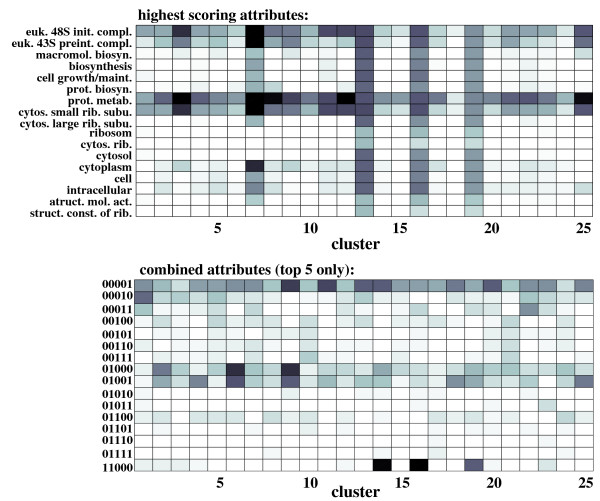
Combinations of GO attributes: Shown is a graphical representation of the contingency tables between the clustering result and the GO annotations. Darker color indicates more genes in that cluster with this annotation. *Upper plot*: The results corresponding to Fig. 2. The highest scoring attributes as determined by the individual mutual information *I*(*C*,*A*_*i*_). The attributes are sorted according to their appearance in the GO database. *Lower plot*: Combined attributes: Shown are the results for the first 5 entries of Table 5. For simplicity, the combinations are given as binary code **A **= (*A*_0_,...,*A*_4_), where *A*_0 _= cytosolic ribosome, *A*_1 _= cytoplasm. *A*_2 _= cell cycle. *A*_3 _= nucleus and *A*_4 _= catalytic activity. Genes not possessing any of the top five attributes listed in Table 5 are omitted.

Most importantly, the selected attributes indeed provide a functional categorization of the obtained clustering, improving the search for significantly enriched annotations. This is visualized in Fig. 3. Shown is a graphical representation of the contingency table between the clustering result (see again Fig. 2 for details) and the combined annotations. As can be observed, the combined annotations provide a more specific functional descriptions of the clustering result. For example, using solely the highest scoring attributes of Fig. 2. genes included in the clusters 13, 16 and 19 are assigned almost uniformly to all selected attributes. However taking only the top five selected attributes of Table 5 into account, several cluster are dominated by specific combinations of attributes, e.g. for cluster 6 genes annotated to 'cytoplasm' (GO:0005737), but to none of the other four attributes, are clearly overrepresented.

## Conclusion

In this work, we have investigated the use of the mutual information as a measure to detect and quantify the interrelation between data generated clusterings and the known functional annotations of genes. Starting with the contribution of individual attributes, we found that the mutual information between a given clustering result and the attributes is highly inhomogeneously distributed. Few attributes show a remarkable overlap with the clustering, while the vast majority of attributes show no, or only little, overlap with the data-driven clustering. These results were robust with respect to parameters in the clustering algorithm, as well as to different choices of datasets.

One of the primary advantages of the mutual information is that it is not restricted to consider only individual attributes contained the GO database. Focusing on combinations of attributes that resulted in a maximal mutual information between the (selected) annotations and the clustering, we demonstrated that this approach extends and enhances the more conventional search for overrepresented attributes in a group of genes of interest. In particular, including only those attributes that further contribute to a characterization of the clustering, in addition to the already selected ones, circumvents the problem of redundant attributes. Within a group of highly dependent attributes, only the one which results in the largest information gain will be selected.

Interestingly, our results indicate that the experimental conditions under which a particular dataset was obtained has no major influence on the top-ranking attributes. For all considered datasets a nearly identical list of highly descriptive attributes was found. Also, these attributes mostly referred to rather abstract functional descriptions, such as 'cell growth', 'catalytic activity', or 'protein metabolism'. This, of course, questions the use of clustering results to gain insight into specific phenomena, such as the transcriptional response to a particular experimental perturbation or knockout experiment. Rather, one usually observes cluster of genes that are known to be tightly co-regulated, such as protein synthesis genes. Only with massive experimental interventions, we must expect the resulting pattern of gene expression to be fundamentally changed and to be directly related to the respective experimental condition.

Finally, it should be emphasized that the application of the mutual information holds a vast potential for further improvements of the method. As yet, we have not focused at predicting a putative functional classification of a specitic cluster. Rather, the mutual information, as used here, represents an average quantity, quantifying the relationship between functional annotation and clusterings as a whole. In this sense, the validity of clustering results can be judged and compared to existing functional annotations. However, the approach tan be straightforwardly extended to detect the prevailing functional annotations of individual clusters, based on the information contained in the GO database.

Along similar veins, the mutual information may also be utilized to further improve the annotation of gene products. Reversing our approach, unknown functional annotations can be predicted based on the available annotations, as well as on membership in a specific cluster. Another advantage of the mutual information in this respect is that, in incorporating additional biological information complementing the GO annotations, it is not restricted to categorical data, but can be extended to include continuous data as well. In particular, the additional information to which a clustering or grouping is compared, is not necessarily restricted to functional annotations. Table 1 may hold any attributes or quantities related to a particular gene or gene product. In this sense, the mutual information constitutes a systematic theoretical basis to investigate the relationship between groups of genes and additional biological information.

## Authors' contributions

RS provided the conceptual backgroung to the analysis and wrote the manuscript. PH performed all computations, including database preparation and clustering. JS participated in in manuscript preparation and supervised the work. All authors read and approved the final version of the manuscript.

## Appendix: Database preprocessing and clustering

Throughout this work, we have used the same preprocessing of the GO database and the gene expression datasets as described in 13: Prior to the evaluation of Eq. (3), the GO attributes were filtered using the following parameters: *i*) No attribute should be shared among almost all genes. All attributes held by more than *N*_max _genes are removed. *ii*) No attribute should be annotated to a single or only a few genes. All attributes that are held by fewer than *N*_min _were removed. *iii*) Redundant attributes should be avoided. To account for this, the normalized pair-wise mutual information (the 'uncertainly coefficient U') was estimated between all attributes. One of each attributes of a pair that had a (normalized) mutual information larger than *U*_max _was removed from the analysis. Note that this step does not fully eliminate the problem of interdependence between the annotation terms. As can be seen later in Fig. 2 and Table 3 the top scoring attributes will still be highly redundant.

The preprocessing removes a large fraction of the attributes. The results were found to be robust with respect to particular choices of (*N*_max_, *N*_min_, *U*_max_). In the following the values *N*_min _= 10, *U*_max _= 0.8, and *N*_max _= max (i.e. no restriction on the maximal number of genes an attribute is assigned to) were used. The clustering of the data was performed using the open source clustering library described in [27]. All results were compared with the algorithms implemented in the software packages matlab and R (, [28]). K-means clustering was chosen in accordance with the results obtained previously in [13]. All scripts used in the analysis are provided as additional files [see additional file 1].

## Supplementary Material

Additional file 1 Source code for data evaluation. The zipped archive [steuer_bmc_SupportMaterial.zip] contains the source code implementing an algorithm to select the Gene Ontology attributes that describe a clustering of expression profiles. Click here for file
